# Supervised categorical principal component analysis for genome-wide association analyses

**DOI:** 10.1186/1471-2164-15-S1-S10

**Published:** 2014-01-24

**Authors:** Meng Lu, Hye-Seung Lee, David Hadley, Jianhua Z Huang, Xiaoning Qian

**Affiliations:** Dept. of Electrical & Computer Engineering, Texas A&M University, College Station, TX 77843 USA; Dept. of Pediatrics, College of Medicine, University of South Florida, Tampa, FL 33620 USA; Dept. of Statistics, Texas A&M University, College Station, TX 77843 USA; Dept. of Computer Science & Engineering, University of South Florida, Tampa, FL 33620 USA

**Keywords:** Categorical Principle Component Analysis, Aggregated Association Analysis, Genome Wide Association Studies

## Abstract

In order to have a better understanding of unexplained heritability for complex diseases in conventional Genome-Wide Association Studies (GWAS), aggregated association analyses based on predefined functional regions, such as genes and pathways, become popular recently as they enable evaluating joint effect of multiple Single-Nucleotide Polymorphisms (SNPs), which helps increase the detection power, especially when investigating genetic variants with weak individual effects. In this paper, we focus on aggregated analysis methods based on the idea of Principal Component Analysis (PCA). The past approaches using PCA mostly make some inherent genotype data and/or risk effect model assumptions, which may hinder the accurate detection of potential disease SNPs that influence disease phenotypes. In this paper, we derive a general Supervised Categorical Principal Component Analysis (SCPCA), which explicitly models categorical SNP data without imposing any risk effect model assumption. We have evaluated the efficacy of SCPCA with the comparison to a traditional Supervised PCA (SPCA) and a previously developed Supervised Logistic Principal Component Analysis (SLPCA) based on both the simulated genotype data by HAPGEN2 and the genotype data of Crohn's Disease (CD) from Wellcome Trust Case Control Consortium (WTCCC). Our preliminary results have demonstrated the superiority of SCPCA over both SPCA and SLPCA due to its modeling explicitly designed for categorical SNP data as well as its flexibility on the risk effect model assumption.

**Electronic supplementary material:**

The online version of this article (doi:10.1186/1471-2164-15-S1-S10) contains supplementary material, which is available to authorized users.

## Introduction

Genome-wide association studies (GWAS) aim to detect the association of genetic variants across the whole genome with traits of interest such as disease phenotypes. They have been successful in identification of susceptibility loci through association analysis of individual single nucleotide polymorphism (SNP) markers with common diseases [1]. Limited by small sample size, however, these analyses are not always reproducible [2, 3]. The associated common variants at the identified susceptibility loci have been found with only modest individual effect [4]. It has always been a challenge for GWAS to detect those SNPs with weak individual effects but may affect disease outcome by strong epistatic effect. In addition, as GWAS focus on single-marker association tests, the obtained results may not provide clear insights into which genes have significant association, how they interact with other genes and/or environment, and what is the underlying disease mechanism. In order to get a better understanding of complex disease, more comprehensive association analysis methods considering interactions among SNPs as well as gene- or pathway-based GWAS have recently attracted researchers' attention [5–7]. For example, multi-locus analysis methods such as multivariate regressions have been proposed to simultaneously test multiple SNPs belonging to a functional region as well as the interactions among them [8]. However, these methods suffer from high degrees of freedom in the statistical tests if a large number of SNPs are simultaneously tested. Alternatively, two groups of aggregated association analysis methods [7, 9–13] focus on testing multiple SNPs with a reduced number of degrees of freedom resulting from: (1) a combined test statistic based on the individual statistical significance for all SNPs; or (2) a combined signal directly derived across all SNPs.

Several popular methods have been proposed to generate a combined test statistic for multiple SNPs/genes for gene- or pathway-based association tests [9, 10, 14–16]. The statistical significance for a gene or pathway is estimated based on its combined test statistic and the corresponding null distribution. Fisher's combination is a simple way to combine *p*-values of all SNPs or genes into a summary statistic determining the gene- or pathway-wise significance [9]. However, the independence assumption may be violated because of linkage-disequilibrium (LD) among SNPs or correlation among genes. Fisher's exact test is another method to aggregate the significance of multiple SNPs or genes in a functional region [17]. Take pathways for example, the test statistic calculates the enrichment of specific significant genes in the given pathway. Similarly, gene set enrichment analysis (GSEA) evaluates association evidence of pathways by calculating and testing an enrichment score of each pathway based on how its constituent genes are ranked by their statistical significance associated with disease [10]. Although these methods have been widely used for pathway-based association analysis, they may lose their power if there are interactions among genes. A potential problem in them is that their combined test statistics are calculated without considering the relationships among SNPs or genes in functional regions of interest.

The second category of aggregated association analysis methods focus on deriving combined signals to aggregate information from multiple SNPs or genes whose joint effect can then be tested by analyzing the relationship between their corresponding combined signal and the trait of interest. The combined signal is mostly generated based on non-linear or linear transformation of individual SNP genotypes to extract the maximum relationship among them [8, 11–13, 18, 19]. Kernel-based approaches typically study all the SNPs in a gene or pathway together based on a kernel function that takes the similarity between individuals while maintaining the relationships among SNPs [11, 12, 20]. Basically, these methods carry out the association analysis by comparing the pairwise genotypic similarity after transformation with the pairwise trait similarity. Test statistics are thus generated with small degrees of freedom based on the adopted kernel functions. Depending on the adopted kernels, these approaches may explore general non-linear interactions among SNPs. In addition to the non-linear modeling of SNP effect based on kernel methods, linear modeling approaches directly combine the original genotypes to summary statistics as combined signals. A key issue for linear approaches is how to choose optimal weights for all the SNPs to derive the combined signal. Prior LD information has been taken advantage of for calculating the weights of SNPs to account for the correlations among SNPs, but it will lose the power if most SNPs only contribute small to moderate effects to the trait of interest, which is often the case as shown in several studies of complex diseases [18, 21]. As a popular linear approach in dimension reduction, principal component analysis (PCA) based methods have been applied for pathway based GWAS [13, 19]. By performing optimal linear combinations of SNP genotypes, it could aggregate SNP effects with optimal weights accounting for their relationships.

Although both PCA based methods and kernel methods could explore the interaction among SNPs for high power in association studies, they share a potential bias since they always have the inherent assumptions that the risk of a SNP is proportional to the number of minor alleles. This bias arises from their data modeling based on the common SNP genotype representation, in which a SNP genotype is represented by numerical values in the domain {0, 1, 2} representing the number of minor alleles for either homozygous or heterozygous allele pairs. However, the induced risk by genotypic mutants may not be directly proportional to the number of minor alleles in a SNP. Instead of the data representation in {0, 1, 2}, it may be more appropriate to represent the SNP data with three different genotypes {00, 10/01, 11}, representing whether we see minor alleles at the corresponding allele pairs. It may not be appropriate to inherently introduce numerical information related to the genetic variation by taking numerical values of this genotype representation as typically done in the existing methods. Therefore, it is more reasonable to analyze SNP data on a premise that SNP data is categorical data except that we have high confidence on the underlying risk effect model, such as commonly adopted additive models. Another challenge with SNP data is the transformation from the quantitative intensity generated during genotyping, into the biologically known underlying number of SNP alleles at a locus. This transformation is performed using calling algorithms, which are specific to genotyping technologies. All subsequent analysis of data, such as GWAS, is dependent on the accuracy and ability of the calling algorithm, several of which were reviewed and improved on by Shah et al [22]. The WTCCC developed an algorithm, CHIAMO, to process their data [23], the results of which are used in our study.

We have previously developed logistic PCA (LPCA) methods [13, 24] for geneand pathway-based analysis of SNP data by explicitly modeling the categorical nature of SNP data. For LPCA, we first transform the genotype data from the domain {0, 1, 2} to binary data {0, 1}, which is assumed to follow a Bernoulli distribution. We have obtained promising results compared with traditional PCA-based SNP analysis that inherently assumes continuous normally distributed SNP data. However, due to the data transformation, LPCA also has an inherent assumption that the risk effect takes either recessive or dominant model. The important information in the original SNP data, especially when we have more general underlying risk effect models, may be lost due to the transformation.

In this paper, we develop a more general PCA denoted as categorical PCA (CPCA) that does not make any specific model assumptions of the effect of genetic mutants on the given trait. We first derive an optimization algorithm for CPCA suitable for categorical data analysis. Similar as conventional PCA, CPCA finds the optimal linear combinations that best explain the observed data but may not derive the principal components that are the most associated with a trait of interest. In order to derive the best principal components capturing the maximum combined effect from multiple SNPs with respect to a given trait of interest, we then apply it in a supervised framework. The best principal components are achieved with the highest correlation with a given trait and are further used in a logistic regression model for association analysis. The supervised framework is similar to the supervised PCA (SPCA) method first proposed for pathway-based gene expression analysis and GWAS based on traditional PCA [19, 25]. By our supervised CPCA (SCPCA), the resulting principal components have the most discriminating power and can be further taken as aggregated predictors for the disease outcome. It ensures that the principal components obtained by CPCA are not deteriorated by noisy SNPs that are irrelevant with the trait. With a more general data model and direct integration of trait information for identifying the most influential SNPs in a functional region, our preliminary results on both simulated genotype data and the Wellcome Trust Case Control Consortium (WTCCC) Crohn's Disease (CD) genome-wide SNP data [23] have demonstrated the advantages of our supervised CPCA over traditional SPCA and supervised LPCA for gene-based and pathway-based aggregated association analysis.

## Methods

Interplaying among disruptions to multiple SNPs or genes has been conjectured to be systems impairments that cause complex diseases, such as cancer and diabetes. In this paper, we develop CPCA to extract optimal combined signals from multiple SNPs without any specific genotype-phenotype model assumptions, which allows more appropriate association analysis of categorical SNP data.

### Principal component analysis

PCA has been implemented in gene expression analysis and GWAS to alleviate the problems in analyzing small sample and high dimensional high-throughput profiling data which is often highly correlated, for example, due to LD. Specifically, PCA finds the orthogonal linear projection that minimizes the mean squared distances from the data points to their low-dimensional projections [26]. Suppose  are the *n* data points and consider that  are their projections in a *l*-dimensional (*l < d*) linear manifold spanned by a basis  with a mean vector ***μ'***. PCA minimizes the following reconstruction error:1

subject to the constraint that  has orthonormal columns. Equivalently, a probabilistic interpretation of PCA assumes that the data points follow a normal distribution with an unknown mean vector ***μ'***. The mean vector, bases, and the corresponding projections can be estimated by maximizing the data likelihood which is an optimization problem equivalent to minimizing (1). Based on the derivation of PCA, it is obvious that PCA is inherently only suitable for continuous variables by making the normal distribution assumption. Therefore, it is not appropriate to directly apply PCA on SNP data which is categorical and does not follow a normal distribution.

### Categorical principal component analysis

It is desirable to develop variants of PCA based on respective modelings for different types of data such as integer, categorical, binary, and nonnegative data. PCA has been extended to the exponential family in previous work [27–29] by assuming data follows a general form of exponential family distributions:2

Here,  is the *i*th data point and ***θ***_*i*_∈ *R*^*d*^ is the "natural parameter" of the corresponding distribution. *G*(***θ***_*i*_) is a function of the form  to ensure that the sum of  over the domain of  equals to 1 and *p*_0_ is a function depending only on . Different members in the exponential family have their respective *G* functions specified in [27], which results in different distributions and different generalization of PCA. To generalize PCA based on the distributions of exponential family, it starts from an important assumption of ***θ***_*i*_ where it is assumed to be a linear combination of bases  with the minimum reconstruction loss represented as . The bases and their corresponding weights  are called as principal component loading vectors and principal component scores respectively. Given the distribution for data points  and the representation of ***θ***_*i*_, the conditional log-likelihood function of the *n* data points with respect to their principal components can be written as:34

where *p*_0_ can be considered as a constant term and ignored here. The principal components resulted from a generalized PCA can then be estimated by maximizing (3). In a special case of the data following a normal distribution, it turns to be the tranditional PCA derived by maximizing this log-likelihood with *G*(***θ***_*i*_) having a form of  where the corresponding parameters are , , and ***μ***. As mentioned earlier, the SNP data in GWAS only has three different genotypes {00, 10/01, 11}. We focus on the derivation of exponential family PCA for categorical data denoted as CPCA in the equivalent categorical domain {0, 1, 2} instead of taking numerical values. For categorical SNP data which follows a multinomial distribution, each observation  is expressed as a set of observation vectors  with only 1 and 0 elements. A 1 or 0 in , *k* ∈ {0, 1, 2} denotes the corresponding outcome equals to *k* or not. Each observation vector  corresponds to a natural parameter vector  determining the success probabilities of the outcomes belonging to category *k*. Each  is projected to a low-dimensional space spanned by its respective basis , sharing the common principal component scores . It can then be represented as . For multinomial distributions, the corresponding *G* function for  is , where ***θ***_*ij*_ is the *j*-th element of ***θ***_*i*_ and c equals to 3 here denoting the number of categories. By substituting this *G* function into (3) and replacing  by the actual parameters , , and ***μ***^*k*^, the log-likelihood function to be maximized for CPCA is rewritten as:56

Where ,  and . ,  and  represent the *i*-th row of , the *j*-th column of  and the *j*-th element of ***μ***^*k*^respectively.

The principal component scores  and principal component loading matrix  could be estimated by maximizing this log-likelihood function with the constraint that  has orthonormal columns. We implement Newton's method for gradient ascent search for the local maximum as the objective function is not jointly concave with respect to , , and ***μ***. Given the objective function (5) with respect to  and , we update  and  by computing their respective first-partial derivative and Hessian matrix for each iteration in Newton's method. Specifically,7

where  represents the updated principal component scores in each iteration;  and  denote the first derivative and Hessian matrix of the objective function *ℓ* with respect to . By basic calculus,  is computed as:89

where  Similarly,  is computed as:10

In each iteration, we also alternatively update  and  based on the following equations:1112

and we have:1314151617

The optimal solution of the corresponding parameters , and ***μ*** can be estimated by the following Algorithm 1. As any non-convex optimization problem, our algorithm is not guaranteed to converge to a global maximum. To overcome the problem of being trapped by local optima, we randomly start the algorithm with different initialization values several times and find the best solution with the maximum likelihood value. The time complexity for this whole procedure is *O*(*ks*^3^) where *s* = *min*(*n*, *d*) and *k* is number of iterations it takes to converge. Specifically, the calculation of the first derivatives and Hessian matrices takes *O*(*dnl*) and *O*(*dnl*^2^) respectively. The update of ,  and ***μ*** takes *O*(*ql*^3^) where *q* = *max*(*n*, *d*). The whole time complexity is mainly determined by QR decomposition procedure which takes *O*(*s*^3^) in each iteration. Our CPCA is in the same magnitude of time complexity as LPCA. Although PCA has a lower time complexity *O*(*nd*^2^ + *d*^3^) if *n > d*, one should be aware that our algorithms are designed for more general risk effect models and may achieve better performance with reasonable sacrifice on running time.

**Algorithm 1** Categorical PCA (CPCA)

1. Initialize with ,  and  by random values. Compute the transpose of .
2. Compute , , , , , and  respectively.
3. Update  by  where each  is updated based on (7) respectively. Compute the *QR* decomposition  and replace  by *Q* for orthonormality constraints.
4. Update  by  where 's are updated by (11) respectively.
5. Update ***μ***^*k*^ by  based on (12) respectively.
6. Repeat steps 2 through 5 until convergence.

### Supervised CPCA for aggregated association analysis

With the principal component scores  derived by CPCA, we can take the first *R* columns of  as the top *R* principal component scores for combined signals from multiple SNPs and  denotes the *j*-th element . For subsequent aggregated association analysis with respect to a trait *y*, the statistical significance for the corresponding SNPs can be estimated by analyzing the association of these derived combined signals with the trait. Specifically, we learn a logistic regression model with the *R* principal components as predictors and *y* as the outcome:18

where  is the posterior probability of the *j*th subject exhibiting *y* given *R* combined signals and the coefficient *β*_*r*_ reflects the joint effect size of a combined signal  on *y*. The statistical significance of multiple SNPs associated with *y* is estimated based on a test statistic . In this paper, we take the first principal component  as the only predictor in the model (18) to estimate the joint effect for simplicity as it contains the largest proportion of information hidden in data. When we take more than one principal components, we may be able to further improve the power when estimating the significant association of multiple SNPs as more information is involved in the model. We will further study this in our future work.

The principal components in CPCA are derived only based on the categorical data distribution with an aim to extract maximum information from the original data, which is not guaranteed to have the most significant association with the trait. Any individual SNP with no correlation with the trait may impair the joint effect estimate in the SNP set to which it belongs since CPCA, as traditional PCA, only focuses on approximating the underlying data distribution. Ideally, we desire a model for aggregated association analysis in which the principal components are derived only based on an optimal SNP subset significantly associated with the trait. However, prior to association analysis, we do not know which SNPs have risk effect on the outcome. In order to solve this problem, a heuristic search procedure is employed to search for an optimal subset of the most significantly associated SNPs with the trait by testing all candidate SNP subsets {*S*_1_, ..., *S*_*v*_} based on the model (18). We set *v* as 20 here. A subset is selected as the optimal subset if its derived principal component scores has the highest discriminating power, which corresponds to the maximum absolute value of the test statistic *t* from (18). For a given SNP set *S*, its test statistic *M* is set as the *t* statistic of its optimal subset, represented as:19

The statistical significance of a SNP set *S* is estimated based on a permutation test of *M* as the distribution of *M* can not be approximated well by any known distribution due to the selection of SNPs.

In summary, based on the categorical data assumption of SNP data, our supervised CPCA takes the following steps to perform aggregated association analysis of a trait for a SNP set *S*:

1. *Generate candidate SNP subsets for a SNP set S*
  For each individual SNP in *S*, its statistical significance reflected by the corresponding *p*-value can be computed by fitting a logistic regression model. Given all SNPs in *S*, we generate 20 incremental candidate subsets by setting 20 thresholds at each increment of 5 percentiles of *p*-values for those SNPs. Hence, 20 subsets of SNPs {*S*_1_, ..., *S*_20_} are formed by sequentially grouping SNPs with *p*-values less than each corresponding threshold.
2. *CPCA on candidate SNP subsets*
  CPCA can be implemented to compute the first PC scores for 20 candidate subsets respectively.
3. *Calculate M statistic for a SNP set S*
  For each candidate subset *S*_*v*_(1 ≤ *v* ≤ 20), we fit the logistic regression model (18) using the corresponding first PC scores and estimate *t*-statistic . Let *M* = {*t*_*v*_: *|t*_*v*_| = *max*_1 ≤ *v* ≤ 20_*|t*_*v*_|}.
4. *Estimate the null distribution of M statistic*
  We perform a permutation test by generating random trait status for each sample from a Bernoulli distribution with the success probability set to the disease prevalence. Based on randomly generated outcomes, the empirical null distribution of *M* statistic can be estimated by repeating steps (1) to (3) and pooled together as a random sample from the null distribution of *M*.
5. *Calculate p-value for a SNP set S*

Given a null distribution of *M* statistic and the *M* value based on true trait, an empirical *p*-value for *S* can be calculated to estimate its significance.

By deriving principal components based on the more general categorical model of the SNP genotype data, CPCA can eliminate the potential bias inherently introduced in PCA and LPCA, In addition, our CPCA is embedded in a supervised framework integrating the trait information for searching and aggregating those relevant SNPs so that we refine the principal components to best discriminate a given trait. With these two methodological contributions, we expect our supervised CPCA can aggregate information accurately from multiple SNPs and achieve higher power in subsequent association analysis by the supervised selection procedure.

## Results

We evaluate our supervised CPCA method by a simulation study for gene-based association analysis as well as pathway-based GWAS for Crohn's disease using WTCCC case-control genome-wide data.

### Simulation experiments

To simulate SNP genotype data with real allele frequencies and linkage disequilibrium (LD) structure patterns, we use the HAPGEN2 [30] simulation tool to generate case and control samples based on a reference set, for which we choose Caucasian cohort (CEU) population on human chromosome 22 from 1000 Genomes project [31]. HAPGEN2 simulates genotype data by resampling this reference set of population haplotypes and an estimate of the fine-scale recombination rate across the region, so that the simulated data has the same LD patterns as the reference data [30]. Unlike other simulation tools simulating a single "disease SNP" on the same haplotype, such as HAPSAMPLE [32], HAPGEN [30], and GWAsimulator [33], HAPGEN2 can simulate multiple SNPs associated with the disease outcome on the same chromosome, which is often the case for many complex diseases [30]. First, we map a total of 6,129 SNPs genotyped with Affymetrix array 6.0 in the chosen reference set to their neighboring genes: SNPs within 5 KB upstream or downstream from a gene are assigned to that gene based on the Ensembl database (Release 67). We randomly select 50 genes with their constituent SNPs as genotyped SNPs for our simulation. These selected genes have 11 to 175 constituent SNPs. Among them, five genes are randomly selected as causal genes for the simulated disease outcome. They contain 56,168,30,12, and 99 SNPs respectively, within which three SNPs for each causal gene are randomly selected as their corresponding disease SNPs respectively. The other 45 genes are considered as null genes with no risk effect on the outcome.

HAPGEN2 models the probability *π*_*i*_ = *P*(*Y*_*i*_ = 1|*G*_*i*_) that subject *i* has disease given SNP genotype *G*_*i*_ ∈ {0, 1, 2}, for which *π*_*i*_ could take three values: *f*_0_, *f*_1_(= *f*_0_ × *rr*_1_), or *f*_2_(= *f*_0_ × *rr*_2_) corresponding to the genotype with different number of minor alleles (*G*_*i*_ = 0, 1, or 2). In this general disease model, *f*_0_, *f*_1_ and *f*_2_ are the corresponding penetrance of the disease and *rr*_1_, *rr*_2_ are the relative risk for heterozygous (*G*_*i*_ = 1) or homozygous ((*G*_*i*_ = 2)) pairs, respectivly. Under a null hypothesis SNP *G*_*i*_ has no effect on disease, *rr*_1_ = *rr*_2_ = 1. To test the power of our supervised CPCA method for detecting causal genes, we studied three different settings for risk effect sizes for disease SNPs in those causal genes. In order to model more general risk effect from different SNPs, we set the homozygote risk for a disease SNP slightly bigger than its corresponding heterozygote risk to avoid any proportional relationship assumptions between its genotype and risk effect size. For example, if we assume a commonly adopted additive model, the relative homozygote risk for a disease SNP is inherently assumed to be equal to the square of its relative heterozygote risk, which may not capture the actual genotype-phenotype relationships in real data. Therefore, we set the relative heterozygote risk and homozygote risk for all disease SNPs at three different levels at (*rr*_1_, *rr*_2_) = (1.2, 1.3), (1.3, 1.4), and (1.5, 1.6). In our simulation study, 500 case and control samples are generated respectively in 100 replicates for each causal gene under different risk levels. The same number of cases and controls are also randomly generated in 100 replicates for 45 null genes. In summary, we simulate 500 (5 × 100) causal genes and 4500 (45 × 100) null genes for each scenario in total.

**Figure 1:**
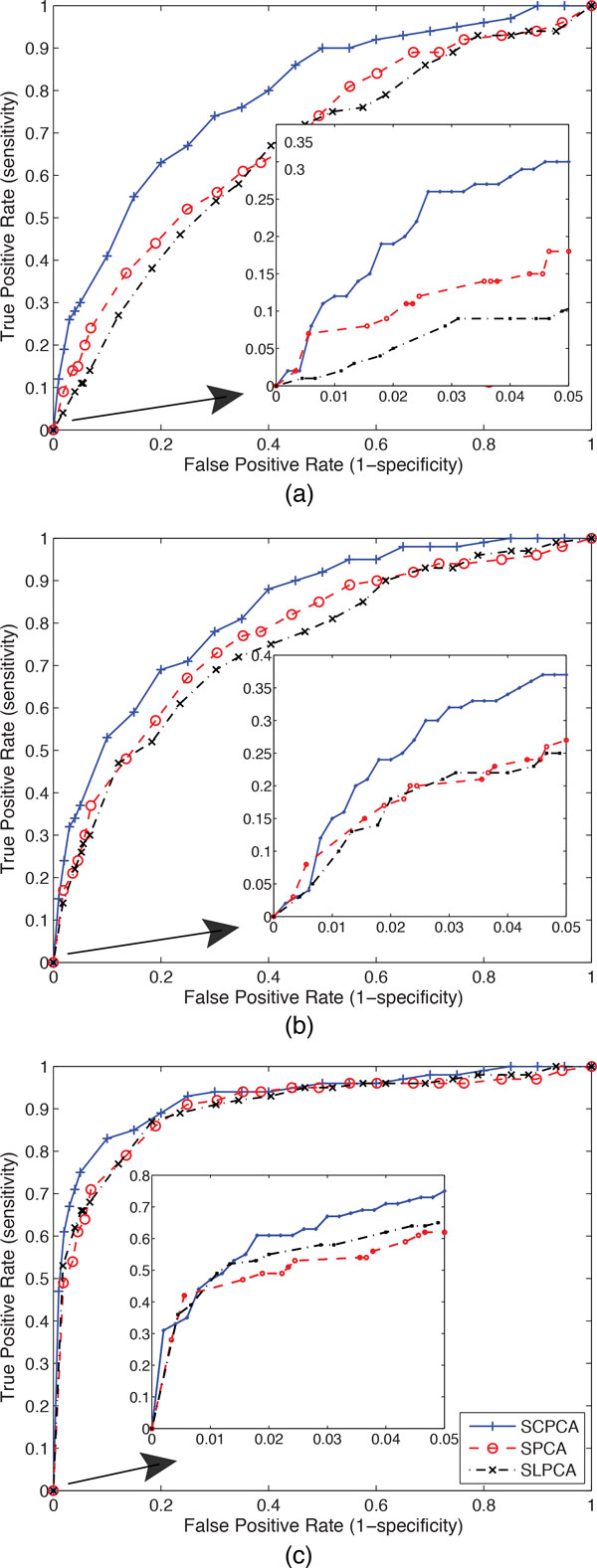
**ROC curves for SCPCA, SPCA, SLPCA at risk level (relative heterozygote risk, relative homozygote risk) = (a) (1.2,1.3); (b) (1.3,1.4); and (c) (1.5,1.6) in gene-based association analysis on simulation data**.

The performance of our supervised CPCA (SCPCA) method on this set of simulated data is evaluated by comparing with the results obtained by SPCA and supervised LPCA (SLPCA) based on two criteria: statistical power and receiver operating characteristic (ROC) curves. The statistical power is computed as the proportion of detected causal genes that are significantly associated with the case-control outcome, for which we have the ground truth as we simulate the outcome based on selected "causal" SNPs. Table 1 provides the statistical power at the significance level 0.05 from different methods, which shows that our method has achieved consistently higher power than the other two methods. Due to explicit modeling of categorical data, our SCPCA performs better than SPCA, which inherently assumes that the data follows a normal distribution. We note that the performance of SLPCA is slightly worse than SPCA in this set of simulation experiments because it loses information when transforming the original categorical genotypes {0, 1, 2} into a binary representation {0, 1} by assuming an inappropriate dominant/recessive model. To further validate the superiority of our SCPCA method, we plot the ROC curves by these three methods for all three risk effect sizes as shown in Figure 1. The ROC curves by SCPCA are always on top of those from SPCA and SLPCA for all scenarios, which demonstrates that its statistical power is consistently higher than the others at different significant levels. In addition, both Table 1 and Figure 1 have illustrated that our SCPCA has achieved more significant performance improvement over the other two methods when the risk effect is small. This demonstrates that SCPCA can perform better due to its explicit modeling of categorical SNP data with more general model assumptions, especially when we have difficult cases where the causal genes are more difficult to detect with smaller risk effect from their constituent disease SNPs. As we expect, based on the results from this simulation experiment, SCPCA is clearly superior to SPCA and SLPCA.

**Table 1:** Comparison of statistical power obtained by SCPCA, SPCA and SLPCA at significance level 0.05 for three risk levels: (relative heterozygote risk, relative homozygote risk) = (1.2,1.3); (1.3,1.4); (1.5,1.6) in gene-based association analysis on simulation data.

Power	Method		
**Risk level**	**SCPCA**	**SPCA**	**SLPCA**
(1.2,1.3)	0.30	0.24	0.14
(1.3,1.4)	0.37	0.37	0.30
(1.5,1.6)	0.75	0.71	0.68

### Analysis for Crohn's disease

We further apply our SCPCA method for a pathway-based association analysis of Crohn's Disease (CD) based on the GWAS case-control data from Wellcome Trust Case Control Consortium (WTCCC) [23]. In this CD dataset, there are 2,005 case samples and 3,004 control samples consisting of 1,504 individuals from the 1958 British Birth Cohort and 1,500 individuals from the UK blood services. After quality control, there are 1,748 cases and 2,938 controls in total with 469,557 SNPs in each sample [23].

To analyze the joint effect from multiple SNPs in functional regions that may be associated with Crohn's disease, we first map all the SNPs in the CD dataset into their corresponding pathways and thus implement SCPCA on each pathway to identify those pathways that are statistically significantly associated with the disease outcome. Specifically, we first download the pathway information from Molecular Signature Database (MSigDB: http://www.broadinstitute.org/gsea/msigdb) and collect two categories of pathways as the prior biology knowledge: C2-CP and C5-BP, corresponding to annotated canonical pathways (CP) from online pathway databases such as KEGG, BioCarta and Reactome pathway databases and GO biological processes (BP), respectively. We further filter out those pathways with more than 250 genes to increase the specificity by avoiding overly broad pathways, which has been similarly done in literature [13, 19]. The resulting 866 CP and 751 BP pathways are taken as candidate functional regions for our aggregated association analysis of Crohn's disease. With the same procedure as in [13], we map SNPs in the preprocessed CD data to these pathways based on the *Homo sapiens* Variation (dbSNP 130) and *Homo sapiens* genes (GRCh37.p7) datasets in the Ensembl database (Ensembl 67) using BiomaRt (http://www.biomart.org/). SNPs are first assigned to their neighboring genes and then mapped to their corresponding pathways according to the previously described pathway information. With the WTCCC CD data and mapped SNPs in all pathways, we implement SCPCA to each pathway and calculate nominal *p*-values from permutation tests. To correct for the multiple-testing issue, we estimate the adjusted *p*-value for each pathway based on the Benjamini-Hochberg method. Significant pathways are identified at false discovery rate level 0.05.

We list 30 representative significantly associated pathways in Additional file 1. Those significant pathways are mostly involved in the following cellular functions: (1) initialization, activation and regulation of transcription factor activity; (2) lipid metabolism or lipid biosynthetic process; (3) regulation of protein kinase activity and protein transport; (4) regulation of cytokine secretion; (5) cellular catabolic process; (6) interleukin production; (7) response to inflammatory and virus; (8) epidermis and muscle development. Many of these pathways are related to the development of human immune system. Their alteration could cause potential malfunctioning of immune system that leads to CD.

To be more specific, those pathways with functions in regulation of cytokine secretion and initialization, activation and regulation of transcription factor are closely related with innate immunity and also have been claimed as statistically significant pathways associated with CD in previous SPCA and SLPCA based analysis [13, 19]. Among these pathways, their common gene NOD2 is the first identified gene associated with CD in previous analysis [34]. It plays an important role in immune response by stimulating immune activity through activating NF-*κ*B. Another common group of causal pathways in these three methods includes gene categories related to response to bacteria and inflammatory. The overly aggressive immune response to bacteria causes inflammatory and is more likely a factor causing CD [35]. Our results also have some other overlap with the previous reported results based on SLPCA [13] in those pathways related with lipid metabolism and interleukin secretion and production including genes: APOA1, IL18, NOD2, CARD8, PYCARD, NLRC4, NLRP12, NLRP3, PYDC1, NLRP2, TLR8 and others. These findings agree well with the recent literature of multiple GWA studies [7, 36, 37]. Substantial alternation of lipid metabolism has been shown in patients with acute CD associated with metabolic disturbances [38]. In addition, our SCPCA found a set of statistically significant pathways related with regulation of protein kinase activity. The mitogen activated protein kinases have been shown with a role in inflammatory bowel disease such as CD by acting as instigative controllers of many signaling pathways regulating the innate and adaptive immune system [39]. We also identified several pathways related with cellular catabolic process and muscle development. Abnormal cellular metabolic process could cause increased energy expenditure, which are typically shown in patients with CD and could further alter muscle mass and function with persist nutritional deficiencies [40]. However, given the fact that there still lacks a complete understanding of the etiology of CD, it is difficult to provide a conclusive evaluation, which will be studied in our future research.

## Conclusions and future work

We have derived CPCA for aggregated association analysis of categorical SNP data, which is further extended to SCPCA in a supervised framework. Our SCPCA captures more relevant information from SNP data based on a better data modelling and aggregates genotypic information from multiple SNPs into a combined signal that is the most associated with the trait by a heuristic selection procedure. By explicitly modeling SNP data as categorical data instead of continuous data with inherent assumptions on numerical effects related with genotypes, our SCPCA has shown higher power compared with SPCA and SLPCA in the gene-based simulation study as well as pathway-based Crohn's disease analysis. On the other hand, SCPCA will lose power if SNP data is indeed under the additive model assumption for introduced risk that affect the trait of interest. When the underlying model is dominant/recessive model or unknown, SLPCA or SCPCA is preferred as they make no assumptions on the numerical effects related with genotypes by assuming SNP data is either binary or categorical. Our future work includes more comprehensive performance evaluation of our SCPCA by comparing with other state-of-the-art methods for association studies based on aggregated statistics including kernel methods [11, 20] as well as hierarchical Bayesian methods [41]. We are also studying new optimization algorithms for more efficient computation, especially when we have large sample size together with millions of SNPs. Another future research direction is to derive methods to simultaneously perform SNP selection when deriving summary statistics by imposing structured sparsity constraints [42].

## Electronic supplementary material

Additional file 1: **Top 30 representative pathways identified by SCPCA in WTCCC Crohn's Disease data set**. This table lists the top 30 statistically significant pathways as well as the number of enriched genes and SNPs for each pathway. Overlapped pathways with those detected by SPCA or SLPCA are also indicated. In the table: The pathways marked as "Yes" have similar functions as the statistically significant pathways detected by SPCA or SLPCA. (PDF 40 KB)
