# Supplementary material for: Supervised categorical principal component analysis for genome-wide association analyses
Source: BMC Genomics. 2014 Jan 24;15(Suppl 1):S10. doi: 10.1186/1471-2164-15-S1-S10 (PMC4046680; doi:10.1186/1471-2164-15-S1-S10)
Supplement: Supplementary file 1 — Additional file 1: Top 30 representative pathways identified by SCPCA in WTCCC Crohn's Disease data set. This table lists the top 30 statistically significant pathways as well as the number of enriched genes and SNPs for each pathway. Overlapped pathways with those detected by SPCA or SLPCA are also indicated. In the table: The pathways marked as "Yes" have similar functions as the statistically significant pathways detected by SPCA or SLPCA. (PDF 40 KB) [file 12864_2014_5683_MOESM1_ESM.pdf]

Pathway	No. of genes	No. of SNPs	Overlap
Peptide metabolic process	10	474	Yes
Lipid biosynthetic process	97	1100	Yes
Transcription initiation	35	279	Yes
Phospholipid biosynthetic process	39	334	Yes
Glycerophospholipid biosynthetic process	30	158	Yes
Lipoprotein metabolic process	33	175	Yes
Membrane lipid biosynthetic process	49	619	Yes
Neuropeptide signaling pathway	14	95	
Steroid hormone receptor signaling pathway	20	177	
Cytokinesis	19	215	Yes
Activation of NF-κB transcription factor	18	261	Yes
Positive regulation of transcription from RNA polymerase II promoter	65	1511	
Cellular carbohydrate metabolic process	122	1610	
Positive regulation of cytokine secretion	10	117	Yes
Positive regulation of transcription factor activity	24	294	Yes
Epidermis development	70	581	
Regulation of DNA binding	47	406	Yes
Positive regulation of binding	28	318	Yes
Interleukin 1 secretion	10	116	Yes
Muscle development	92	1979	
Cellular catabolic process	209	2076	
Cellular lipid catabolic process	34	236	
Intracellular protein transport	139	1545	
Regulation of protein kinase activity	151	1393	Yes
Glycoprotein metabolic process	88	1466	
Regulation of transcription factor activity	40	375	Yes
Interleukin 8 production	11	74	Yes
Inflammatory response	124	1146	Yes
Positive regulation of cell proliferation	142	1991	Yes
Protein targeting	104	1202	
Response to virus	49	313	Yes
